# Impact of intravitreal injection therapy on contrast sensitivity in patients with nAMD and DME

**DOI:** 10.1007/s00417-022-05944-8

**Published:** 2023-01-09

**Authors:** Sebastian Dieke, Stefanie Wurche, Anne Ruebsam, Christopher Wirbelauer, Antonia M. Joussen, Sibylle Winterhalter

**Affiliations:** 1 Beuth Hochschule for Technology Berlin, University of Applied Sciences, Berlin, Germany; 2 Department of Ophthalmology, Charité-University Medicine Berlin, Corporate Member of Freie Universität Berlin, Humboldt-Universität zu Berlin and Berlin Institute of Health, Campus Virchow-Klinikum, Augustenburger Platz 1, 13353 Berlin, Germany; 3 Berlin Institute of Health (BIH), Berlin, Germany; 4 Eye Clinic Berlin-Marzahn, Berlin, Germany

**Keywords:** Contrast sensitivity, Mars Letter Contrast Sensitivity Test, Freiburg Visual Acuity and Contrast Test, Neovascular age-related macular degeneration, Diabetic macular edema, Intravitreal injection

## Abstract

**Purpose:**

The study aims to evaluate changes in contrast sensitivity (CS) during therapy with intravitreal vascular endothelial growth factor (VEGF) inhibitors in patients with neovascular age-related macular degeneration (nAMD) and diabetic macular edema (DME).

**Methods:**

Prospective, uncontrolled, multicenter study on patients with neovascular AMD or DME who underwent intravitreal injection therapy with Ranibizumab, Aflibercept, or Bevacizumab was conducted. Best corrected visual acuity (BCVA) and CS measured by Mars Letter Contrast Sensitivity Test (MLCS) and Freiburg Visual Acuity and Contrast Test (FrACT) in logCS were evaluated before 3 consecutive VEGF inhibitor injections, which followed the pro renata regimen in treatment-naïve and pretreated eyes with a maximum of 9 injections. Correlation of MLCS and FrACT was calculated by the Spearman’s rank correlation coefficient.

**Results:**

Eighty eyes of 74 patients (mean age 72.7; SD ± 9.96) were included. BCVA improved significantly from 0.44 (SD ± 0.21) logMAR to 0.38 (SD ± 0.23) logMAR by 0.06 (SD ± 0.14) logMAR values (*p* < 0.001). CS measured by MLCS increased significantly from 1.27 (SD ± 0.25) logCS to 1.39 (SD ± 0.22) logCS (*p* < 0.001). CS measured by FrACT also improved significantly from 1.22 (SD ± 0.32) logCS to 1.30 (SD ± 0.29) logCS (*p* = 0.035). A positive correlation between MLCS and FrACT was found (*r* = 0.389; *p* < 0.001). Despite statistical significance, results for BCVA, MLCS, and FrACT failed clinical significance. Overall best test results were achieved with MLCS.

**Conclusions:**

Intravitreal injection therapy with VEGF inhibitors led to an improvement of BCVA and CS measured by MLCS and FrACT. MLCS was superior and more sensitive compared to FrACT and even BCVA to evaluate CS in elderly patients with macular pathology.

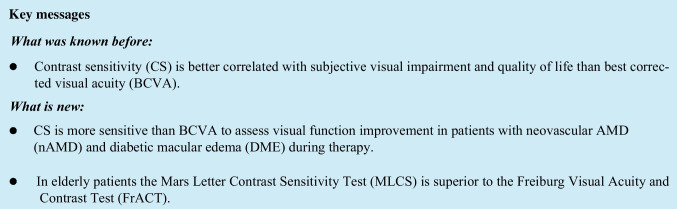

## Introduction

Best corrected visual acuity (BCVA) is the most commonly used test to assess visual function in routine clinical practice. However, BCVA only describes one quality of visual function and is not sufficient enough to differentiate objects from their surroundings. Contrast sensitivity (CS) is a further important factor, as objects of our daily life do have a multiplicity of sizes and contrasts. As such, an impaired visual function combined with a decrease in CS and depth perception is strongly associated with falling in elderly people [1].

Contrast is a physical measure, which describes the difference of the luminance of a brighter area and an adjacent darker area and can be calculated by different formulas like the Michelson contrast or the Weber contrast [2]. CS describes the inverse value of the visual contrast threshold. The visual contrast threshold is the slightest cognizable contrast between 2 parallel or 2 consecutive seen objects with different luminance. For clinical studies the logarithm of the CS value is most commonly used. CS also depends on the physiological characteristics or abnormalities of the eye, for example, corneal scars or haze, previous history of refractive surgery, cataract, vitreous haze, age-related macular degeneration, diabetic retinopathy, glaucoma, retinitis pigmentosa, optic neuritis, or systemic diseases like multiple sclerosis, Parkinson’s disease, or dementia. In addition, CS may be influenced by the used optotypes because CS for letters or Landolt circles is smaller than for sine-waves.

The Michelson contrast is described by *C*_M_ = (luminance_max_ − luminance_min_) / (luminance_max_ + luminance_min_) and is especially used for sine-waves or surfaces with different luminances but same sizes [2]. Whereas the Weber contrast is described by *C*_w_ = (luminance_max_ − luminance_min_) / luminance_max_ or (luminance background − luminance target optotype) / luminance background and is especially used for small optotypes on a uniform background [2].

CS can be measured with different test charts, insight devices, and computer-based tests including the Mars Letter Contrast Sensitivity Test (MLCS) [3, 4] and the Freiburg Visual Acuity and Contrast Test (FrACT) [5].

The main difference between both tests are the optotypes used. Some CS tests use sine-waves, while others use Landolt circles, numbers, or letters [2]. For example, the Vistech and FACT charts, the Vector Vision CSV-1000E chart, and the Cambridge Contrast charts contain sine-waves [2]. CS tests with optotypes can be subdivided into CS tests with varying contrast or CS tests with constant contrast [2]. The Pelli-Robson chart and the MLCS are examples for CS tests with contrast varying across letters. The Pelli-Robson chart consists of a 60 cm × 80 cm sized table with sloan letters, which are arranged in 8 lines. Each line contains 6 sloan letters and the contrast reduces from 3 sloan letters, which form a triplet, to the following 3 sloan letters [2].

The MLCS also consists of a test chart with sloan letters and is similar to the Pelli-Robson chart. In contrast, CS measured with the MLCS reduces from letter to letter and thus can be graduated more subtle than using the Pelli-Robson chart [2].

The Low-Contrast Bailey Lovie charts and the LCS test of Buser are examples for CS tests with constant contrast [2]. The Low-Contrast Bailey Lovie charts are similar to ETDRS charts. The LCS test of Buser contains test charts with Landolt circles of high contrast on the front side and low contrast on the back side.

Different CS tests were also developed for children, for example, the Hidden Heidi test or the LEA Low-Contrast Symbol Charts [2].

The FrACT is a free available software for visual acuity and CS evaluation with Landolt circles as suitable optotypes (as prescribed in DIN EN ISO 58220) and can be tested on a computer screen (FrACT; http://michaelbach.de/fract/) [6–8].

In industrialized countries with an increased average life expectancy the main causes of visual impairment are neovascular age-related macular degeneration (nAMD), diabetic retinopathy (DR), and glaucoma [9, 10]. In early stages of diabetic retinopathy, a decrease in CS can be detected even if visual acuity is not yet affected [11, 12]. Likewise CS might be reduced in AMD before visual changes have become apparent [13, 14].

Despite its usefulness CS measurements were not included during the Ranibizumab [15–19] and Aflibercept [20, 21] approval trials for the treatment of nAMD and DME as well as in the head-to-head trial of Ranibizumab versus Bevacizumab for nAMD [22, 23]. Therefore, the aim of our study was to evaluate short-term changes in CS after therapy with intravitreal vascular endothelial growth factor (VEGF) inhibitors in nAMD and DME and to compare the sensitivity of the two different CS testing methods MLCS and FrACT.

## Material and methods

This prospective, multicenter study was conducted at 4 sites between March and October 2015. Patients with nAMD or DME under therapy with either Ranibizumab, Aflibercept, or Bevacizumab were included into the study if BCVA was measured above 1.3 logMAR so that the potential of visual improvement was given. Injection therapy followed the pro renata regimen of the IVAN trial with 3 consecutive injections [23]. At the beginning of the study we intended to include only treatment-naïve patients, but the number of eligible patients was too low, so that we widened the inclusion criteria to patients with ≤ 9 injections of VEGF inhibitors before baseline. Exclusion criteria were ≥ 9 injections of a VEGF inhibitor before baseline, history of corneal refractive surgery or pars plana vitrectomy, any corneal abnormality (e.g., dystrophy, scar, haze) or ocular infection or inflammation, and history of medical use of substances with known impact on CS (e.g., anti-depressants, cytostatic drugs, anti-rheumatic agents).

Main outcome measure was to evaluate changes in CS after therapy with intravitreal VEGF inhibitors and to compare 2 different tests for CS, the MLCS and FrACT. We utilized the MLCS test instead of Pelli-Robson charts because the MLCS test is easier to carry out at a uniform illumination. No additional optical coherence tomography (OCT) images were taken to correlate CS changes with changes in fluid accumulation or central retinal thickness (CRT).

All patients gave their informed consent prior to inclusion into the study and all procedures were in concordance with the tenets of the Declaration of Helsinki. The study was approved by the local ethics committee of the Charité-University Medicine Berlin (EA4/072/22).

### Examinations

All measurements were performed by 2 experienced optometrists (SD and StW) under standardized conditions with a room illumination of 90 cd/m^2^.

The LS 100 measurement device (Konica Minolta) was used to check the luminance at the 4 different study sites. MLCS and FrACT were performed in a dark room, with a standardized illumination of 100 cd/m^2^ of the MLCS charts and 70 cd/m^2^ around the notebooks. BCVA, MLCS, and FrACT were performed at baseline and after 4 and 8 weeks before 3 consecutive VEGF inhibitor injections in patients with nAMD and DME with ≤ 9 injections of anti-VEGF therapy. The forced-choice principle was used. BCVA, MLCS, and FrACT testing were performed within approximately 15 min to avoid inattention of the patients.

### BCVA

BCVA was measured in logMAR using ETDRS charts or in decimal using a visual acuity projection system at a background luminance of 120–140 cd/m^2^. Decimal values were transformed into logMAR using the Freiburg visual acuity conversion chart [39]. Visual acuity testing was always started at a BCVA of 1.3 logMAR to check the inclusion criterion of minimum BCVA. Head tilting was allowed in patients with difficulties of central fixation due to macular pathology.

### MLCS

The MLCS test (The Mars Letter Contrast Sensitivity Test. Form 1. © 2003–2004, Mars Perceptrix Corporation), consists of 3 test charts with 48 sloan letters (C, D, H, K, N, O, R, S, V, Z) and a continuous letter by letter reduction of CS with a difference of 0.04 logCS between the consecutive letters. The MLCS test starts with 0.04 logCS and covers a CS of 91–1.2%. A luminance of 100 cd/m^2^ at the middle of the test charts was chosen, as a luminance of 60–120 cd/m^2^ is recommended for the test. Testing was carried out at a distance of 50 cm (1.4 logMAR equivalent) and the procedure followed the manufacturer’s instruction. The test was stopped after 2 consecutive errors. The final score was the logCS of the last correctly read letter, minus 0.04 logCS for any errors made before.

During this study the Weber contrast was used because it is more specific for small dark objects than the Michelson contrast: *C*_w_ = (luminance background − luminance target optotype) / luminance background. The Weber contrast was log transformed in logCS, which is most commonly used.

### FrACT

The FrACT version 3.9.1 was used for this study. A distance of 50 cm and a Landolt circle diameter of 125′ were chosen for intertest comparability to the MLCS test. The 8 alternatives of the Landolt circles were reduced to 4 alternatives for a better compliance of the elderly patient cohort leading to a probability of guessing of 25%. The testing procedure started always with a CS of 0.74 logCS (18.2%). The FrACT utilizes the best parameter estimation by a sequential testing (PEST) algorithm for threshold determination. Test results were documented by the investigator to avoid adaptation modifications by the study patient. Every examination consisted of 24 Landolt circles as recommended and 3 easy trials were included to encourage the patients. A presentation time of 60 s was used. The FrACT was conducted on 2 notebooks (Lenovo ideapad S2015; 11.6-inch LCD display; 1366 × 768-pixel resolution; 32-bit color depth and Acer Aspire One; 10.1-inch LCD display; 1024 × 600-pixel resolution; 32-bit color depth). A calibration of both monitors was performed at baseline with the professional tool i1display pro and the i1profiler software (xRite). This system allows automatic determination of the luminance of the monitor and surroundings and to carry out *γ* corrections of the notebooks. The notebooks were switched on 30 min before each examination to avoid luminance variations. The patients had to look in a right angle on the monitor during every FrACT examination because illumination and contrasts are dependent on the sight angle. CS classification for MLCS and FrACT is shown in Table 1.

**Table 1:** CS classification

CS in logCS	MLCS	FrACT (SD)
Normal:		1.82 (± 0.11) [40] 1.51 (± 0.1) [41] 2.35 (± 0.22) [42]
Young adults > 60 years old	1.72–1.92 1.52–1.76	
Cataract patients		1.86 (± 0.24) [42]
Reduced:		
Moderate Severe Extremely	1.04–1.48 0.52–1.0 < 0.48	

### Statistics

Statistics were performed with SPSS (Version 21.0.0.0, IBM, USA) and Microsoft Excel 2010 in cooperation with the Institute for Biometry and Clinical Epidemiology of the Charité-University Medicine Berlin. The matched paired Wilcoxon signed rank test was used to evaluate BCVA and CS changes between baseline and the 4- and 8-week visits. Correlation of MLCS test and FrACT was calculated with the Spearman’s rank correlation coefficient. The results are expressed in mean ± standard deviation (SD) and were regarded as statistically significant if *p* was below 0.05.

## Results

Eighty-six patients (92 eyes) were included into the study after written informed consent was obtained between March and October 2015. Twelve eyes of 12 patients had to be excluded of the final analysis because of delayed appointments (8 patients), missing compliance (2 patients), status post pars plana vitrectomy (1 patient), and status post YAG capsulotomy (1 patient) during follow-up. In conclusion, 74 patients (80 eyes) were included in the final analysis. Table 2 shows the baseline characteristics of the 80 study eyes.

**Table 2:** Baseline demographics

	Total	nAMD	DME
Eyes, *n*	80	49 (61%)	31 (39%)
Gender, *n* (%)			
Female (41 patients) Male (33 patients)	44 (55) 36 (45)	33 (41.25) 16 (20)	11 (13.75) 20 (25)
Mean age in years (SD) Female Male	72.7 (9.96) 72.9 (10.97) 72.4 (8.63)	76.8 (6.5) 76.4 (7.3) 77.2 (5.7)	65.5 (11.65) 62.9 (14.5) 68 (8.8)
Treatment, *n* (%)			
Naïve Pretreated	40 (50.00) 40 (50.00)	27 (55.10) 22 (44.90)	13 (41.94) 18 (58.06)
Lens status, *n* (%)			
Phakic Pseudophakic	37 (46.25) 43 (53.75)	23 (46.94) 26 (53.06)	14 (45.16) 17 (54.84)
Mean BCVA in logMAR (SD)	0.44 (0.21)	0.45 (0.21)	0.42 (0.20)
Mean CS in logCS (SD) of MLCS	1.27 (0.25)	1.24 (0.26)	1.3 (0.22)
Mean CS in logCS (SD) of FrACT	1.22 (0.32)	1.16 (0.32)	1.32 (0.28)

Of the 74 patients with a mean age of 72.7 (SD ± 9.96) years, 41 patients (55%) were female and 33 patients (45%) were male. Both eyes were treated in 3 female and 3 male patients. In these 6 patients (8.1%) both eyes were included into the study. Forty-nine eyes (61%) had nAMD and 31 eyes (39%) diabetic macular edema (DME). The nAMD group consisted predominantly of female patients and the DME group predominantly of male patients (Table 2). Patients with DME (mean age of 65.5 years; SD ± 11.65) were on average 10 years younger than patients with nAMD (mean age of 76.8 years; SD ± 6.5).

Intravitreal injections were performed with Ranibizumab in 42 eyes (52.5%), with Bevacizumab in 20 eyes (25%), and with Aflibercept in 18 eyes (22.5%). The study group consisted of 40 (50%) treatment-naïve and 40 (50%) pretreated eyes. Eyes with nAMD were treatment naïve in 27 (55.1%) eyes and pretreated in 22 (44.9%) eyes. Eyes with DME were treatment naïve in 13 (41.94%) eyes and pretreated in 18 (58.06%) eyes. A phakic lens status was documented in 37 (46.25%) eyes and 43 (53.75%) eyes were pseudophakic.

### BCVA

At baseline 56 eyes (70%) had a BCVA between 0.6 logMAR and 0.3 logMAR. Mean BCVA improved significantly from 0.44 (SD ± 0.21) logMAR at baseline to 0.38 (SD ± 0.23) logMAR before the 3rd injection (*p* < 0.001) (Table 3). Subgroup analysis demonstrated a greater improvement in BCVA in eyes with DME from 0.42 (SD ± 0.2) logMAR at baseline to 0.35 (SD ± 0.17) logMAR (*p* < 0.001) (Table 3) compared to eyes with nAMD with an improvement from 0.45 (SD ± 0.21) logMAR at baseline to 0.4 (SD ± 0.25) logMAR (*p* = 0.007) (Table 3). Further subgroup analysis revealed a statistically significant increase in BCVA after Ranibizumab (*p* < 0.001) and Bevacizumab (*p* = 0.006) injections, but not after Aflibercept (*p* = 0.35) (Table 3).

**Table 3:** Course of BCVA, MLCS, and FrACT during therapy with 3 consecutive VEGF inhibitor injections

	BCVA in mean logMAR (SD)			MLCS in mean logCS (SD)			FrACT in mean logCS (SD)		
	Total	nAMD	DME	Total	nAMD	DME	Total	nAMD	DME
*V*_1_	0.44 (0.21)	0.45 (0.21)	0.42 (0.20)	1.27 (0.25)	1.24 (0.26)	1.3 (0.22)	1.22 (0.32)	1.16 (0.32)	1.32 (0.28)
*V*_2_	0.41 (0.23)	0.42 (0.25)	0.38 (0.21)	1.33 (0.24)	1.29 (0.26)	1.4 (0.19)	1.26 (0.26)	1.18 (0.25)	1.38 (0.23)
*V*_3_	0.38 (0.23)	0.40 (0.25)	0.35 (0.17)	1.39 (0.22)	1.36 (0.23)	1.44 (0.2)	1.3 (0.29)	1.25 (0.31)	1.38 (0.23)
Improvement *V*_1_–*V*_3_ (*n* = 80 eyes)	− 0.06 (0.14)	− 0.05 (0.17)	− 0.07 (0.09)	0.13 (0.2)	0.12 (0.19)	0.13 (0.21)	0.07 (0.27)	0.09 (0.29)	0.06 (0.26)
*p*-value *V*_1_–*V*_3_	< 0.001*	0.007*	< 0.001*	< 0.001*	< 0.001*	< 0.001*	0.035*	0.047*	0.3
Improvement *V*_1_–*V*_3_ Ranibizumab (*n* = 42 eyes)	− 0.09 (0.14)			0.14 (0.23)			0.08 (0.27)		
*p*-value *V*_1_–*V*_3_ Ranibizumab	< 0.001*			< 0.001*			0.046*		
Improvement *V*_1_–*V*_3_ Bevacizumab (*n* = 20 eyes)	− 0.06 (0.09)			0.14 (0.19)			0.06 (0.35)		
*p*-value *V*_1_–*V*_3_ Bevacizumab	0.006*			0.004*			0.9		
Improvement *V*_1_–*V*_3_ Aflibercept (*n* = 18 eyes)	− 0.01 (0.18)			0.09 (0.12)			0.08 (0.2)		
*p*-value *V*_1_–*V*_3_ Aflibercept	0.35			0.013*			0.2		

*V*_*1*_: baseline, 1st injection; *V*_*2*_: 2nd injection; *V*_*3*_: 3rd injection

^*^Statistically significant improvement

### MLCS

**Fig. 1:**
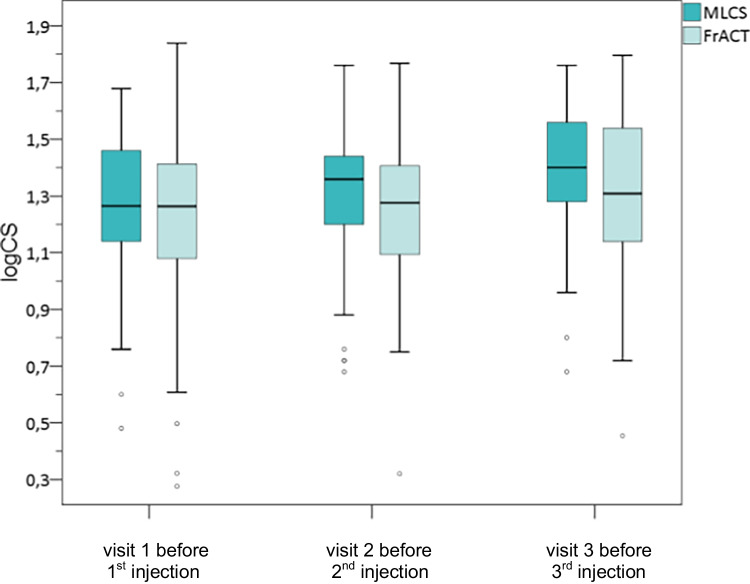
Comparison of MLCS and FrACT during therapy with 3 consecutive VEGF inhibitor injections

**Fig. 2:**
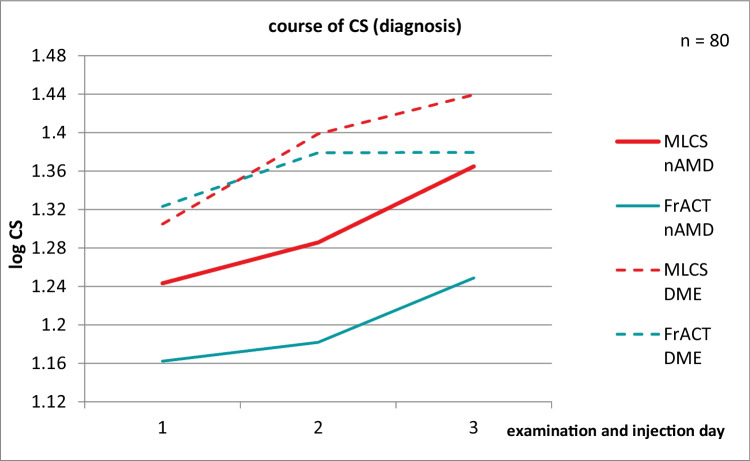
Course of CS measured with MLCS and FrACT in patients with nAMD and DME

In accordance with the improvement in BCVA mean MLCS improved significantly from 1.27 (SD ± 0.25) logCS at baseline to 1.39 (SD ± 0.22) logCS before the 3rd injection (*p* < 0.001) (Table 3; Fig. 1). Subgroup analysis further showed a similar statistical significant MLCS improvement in eyes with nAMD from 1.24 (SD ± 0.26) logCS at baseline to 1.36 (SD ± 0.23) logCS (*p* < 0.001) and DME from 1.3 (SD ± 0.23) logCS at baseline to 1.44 (SD ± 0.2) logCS before the 3rd injection (*p* < 0.001) (Fig. 2). A statistical significant improvement was also seen in eyes treated with Ranibizumab (*p* < 0.001), Bevacizumab (*p* = 0.004), and Aflibercept (*p* = 0.013) (Table 3).

### FrACT

Mean FrACT improved significantly from 1.22 (SD ± 0.32) logCS at baseline to 1.3 (SD ± 0.29) logCS (*p* = 0.035) (Table 3; Fig. 1). Subgroup analysis only demonstrated a statistical significant improvement in FrACT values in nAMD patients from 1.16 (SD ± 0.32) logCS at baseline to 1.25 (SD ± 0.31) logCS before the 3rd injection (*p* = 0.047) (Fig. 2) and after Ranibizumab therapy (*p* = 0.046) (Table 2). FrACT improved in patients with DME (*p* = 0.3) (Fig. 2) and after therapy with Bevacizumab (*p* = 0.9) and Aflibercept (*p* = 0.2), but without statistical significance (Table 3).

### MLCS versus FrACT

**Fig. 3:**
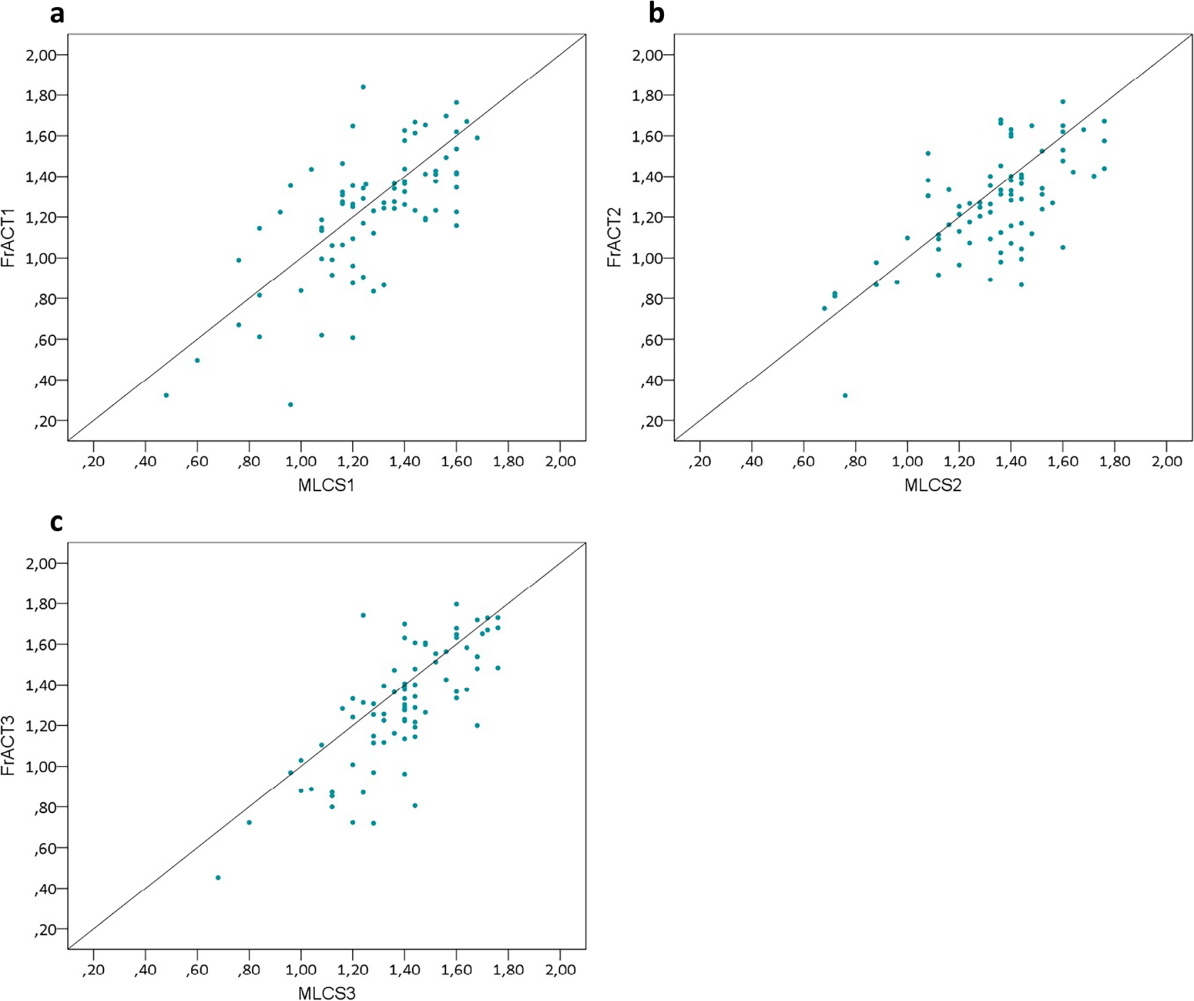
**a**–**c** Correlation between MLCS testing and FrACT calculated with Spearman’s rank correlation coefficient for visit 1, visit 2, and visit 3

A positive but moderate correlation between MLCS testing and FrACT was found through Spearman’s rank correlation coefficient (0.389; *p* < 0.001) (Fig. 3a–c).

Patients evaluated MLCS charts as predominantly positive because the testing procedure is similar to BCVA testing. In contrast to MLCS, which starts with 0.04 logCS, FrACT starts with 0.74 logCS. Thus, patients with decreased BCVA could not see the first Landolt ring of the FrACT directly at the beginning of the examination, leading to confusion in these patients.

## Discussion

The aim of our study was to evaluate short-term changes in CS in patients with nAMD and DME that underwent anti-VEGF therapy. In addition, the practicability of the MLCS test, which consists of 3 test charts and the FrACT, which is a computer-based test, was examined.

Overall our results showed comparable gains of BCVA, MLCS, and FrACT in patients with nAMD. In patients with DME BCVA and CS assessed by MLCS were able to detect statistically significant improvements, whereas FrACT was less sensitive. Further subgroup analysis revealed favorable results for Ranibizumab-treated eyes (52.5%) with comparable gains in BCVA, MLCS, and FrACT. The smaller Bevacizumab-treated group (25%) achieved improvements in BCVA and CS assessed by MLCS but not by FrACT. In the smallest subgroup of Aflibercept-treated patients (22.5%) only MLCS testing was able to detect a significant treatment effect. Thus, the most sensitive test to detect an improvement in visual function after anti-VEGF therapy appears to be MLCS. Spearman’s rank correlation coefficient showed a positive but moderate correlation between MLCS and FrACT testing as seen in Fig. 3a–c. The better results achieved by MLCS testing in comparison to FrACT are consistent with the patients’ preference for MLCS testing. Most patients felt more comfortable with MLCS testing than FrACT because the test strategy for FrACT was more frustrating in patients with decreased BCVA who could not see the first Landolt circle at 0.74 logCS.

In our study 3 consecutive anti-VEGF injections led to a statistical significant improvement in BCVA of − 0.6 logMAR (*p* < 0.001) which equals a BCVA gain of approximately 3 ETDRS letters, an improvement which fails to be clinically meaningful. This is due to the fact that 50% of patients were not treatment-naïve at baseline. During the MARINA and ANCHOR [15, 16] trials on treatment-naïve nAMD patients, the greatest BCVA gains were achieved after the 1st and 2nd Ranibizumab injections. Thereafter, BCVA raised only marginally and was mainly maintained. Thus, the lack of BCVA improvement in our study is in line with the literature. Moreover, we demonstrated a greater sensitivity of CS testing with MLCS compared to BCVA in detecting differences in vision after anti-VEGF therapy in patients with nAMD and DME.

Bellmann et al. [24] demonstrated the natural course of BCVA and CS measured with Pelli-Robson charts in nAMD patients and found a moderate correlation between BCVA and CS. It was observed that a 6-letter loss of CS corresponds to a deterioration of 0.15 logCS and to a 15-letter loss on the ETDRS chart [24–26].

Our results are comparable to the results of Nixon et al. [27], who evaluated CS with Pelli-Robson charts in nAMD patients after a therapy switch from Ranibizumab to Aflibercept. CS improved around 0.08 logCS with statistical significance (*p* < 0.001), whereas BCVA did not change.

Pelli-Robson charts are similar to MLCS charts [3, 28], which were used in our study. In our nAMD patients CS measured with MLCS charts improved significantly with 0.12 logCS (*p* < 0.001) and BCVA with 0.05 logMAR (*p* = 0.007).

Higher BCVA and CS gains can be expected in treatment-naïve nAMD patients as seen in the study of Munk et al. [29]. Over the study period of 1 year an improvement in BCVA (*p* = 0.11), CS (*p* < 0.0001), and in microperimetry (*p* < 0.0001) was detected in patients treated with monthly injections. CS was measured with Pelli-Robson charts and patients gained 6.4 CS letters, which is equivalent to a mean gain of 0.17 logCS [29]. Given the fact that we included 44.9% pretreated nAMD patients, a gain of 0.12 logCS in our study cohort is comparable to the results of Munk et al. [29].

Another study of pretreated nAMD patients with bilateral involvement demonstrated that CS followed by binocular maximum reading speed showed a better correlation (*r* = 0.59; *p* < 0.0001) with the near subscale score of Quality of Life (QoL) assessment than BCVA [30].

It is well known that BCVA measures only the spatial resolution of the visual system with black letters on white background in a 100% contrast environment [31–33]. Opposed to that, CS measures the threshold contrast to detect the presence of minimal luminance differences between areas or objects [31–33]. Thus, CS could be a valuable tool to quantify vision-related QoL in clinical trials on nAMD patients [30] because patients might appreciate changes of CS more than of visual acuity.

Different studies examined the correlation of CS and optical coherence tomography (OCT) findings [34, 35].

Keane et al. studied 122 treatment-naïve nAMD patients [34]. CS was measured by Pelli-Robson charts and the strongest correlation was found between decreased CS and an increase in subretinal fibrous scar tissue (*r* =  − 0.4944, *p* < 0.001). Decreased BCVA, measured with ETDRS charts, was also correlated with increased central retinal thickness (CRT) at the foveal center (*r* =  − 0.4530; *p* < 0.001) [34].

Another study by Sato et al. showed a strong correlation between loss of fundus autofluorescence, CS (*p* < 00.1), BCVA (*p* < 0.001), and reading speed (*p* < 00.1) in pretreated patients with nAMD [35]. This seems logical, because retinal pigment epithelium (RPE) function is mandatory connected to visual function. Their work also underlines the importance of measuring CS as a quality of visual function in patients with nAMD.

Even in patients with mild nonproliferative diabetic retinopathy without history of DME, CS was reduced significantly and associated with outer retinal thickness reduction measured by spectral domain (SD)-OCT [36]. These results highlight the promising role of CS measurements in diabetic patients in detecting early neurodegenerative changes.

A direct comparison to our study is not possible, because our patients did not receive OCT images during a therapy cycle of 3 consecutive VEGF inhibitor injections. A comprehensive ophthalmologic examination including OCT assessment was performed 4 weeks after the 3rd injection.

Another study showed a moderate but statistically significant correlation between CS and MP in patients with DME without significant correlation to CRT assessed by SD-OCT [31]. In contrast, BCVA was significantly correlated to CS, MP, and CRT.

Our results are comparable to another study by Nixon et al. [37], who evaluated CS, BCVA, and CRT after therapy switch from Ranibizumab to Aflibercept in recalcitrant DME [37]. Mean CS increased with 0.06 logCS (*p* < 0.001) and BCVA with − 0.05 logMAR at week 20 (*p* = 0.0016). In our DME patients CS, measured with MLCS charts, improved with 0.13 logCS (*p* < 0.001) and BCVA with − 0.07 logMAR at week 8 (*p* < 0.001).

Subgroup analyses of the RIDE and RISE studies on Ranibizumab for DME included CS testing with Pelli-Robson charts [38]. Patients with a 3-step improvement of diabetic retinopathy severity score (DRSS) experienced an improvement of + 15.1 ETDRS letters and 0.125 logCS. Although our DME patients did not gain 3 ETDRS lines, the logCS gain of 0.13 logCS was comparable. This underlines the importance of CS testing as a quality of visual function.

There are several limitations to our study. All the above-mentioned studies used Pelli-Robson charts, which are best comparable to the MLCS test charts, used in our study, because the literature on MLCS and FrACT testing for nAMD and DME is scarce. Therefore, our results are comparable to the above-mentioned literature to a limited extend. In addition, our patients did not receive OCT images during a therapy cycle of 3 consecutive anti-VEGF injections and only 40 (50%) eyes were treatment-naïve. Further, it has to be stated that test results for BCVA, MLCS, and FrACT demonstrated a statistically significant improvement, but did not lead to a clinically meaningful improvement.

A major strength of our study is the fact that we demonstrated CS changes during therapy with VEGF inhibitors in patients with nAMD and DME with two different methods of CS testing. In addition, we evaluated the practicability of the two different methods for CS testing. Herein we found MLCS testing to be more easy to perform from the patients’ perspective.

In conclusion, our study demonstrates that intravitreal injection therapy with VEGF inhibitors led to a statistical significant improvement in BCVA and CS, measured by MLCS and FrACT. In elderly patients with macular pathology MLCS testing was superior and more sensitive compared to FrACT and even BCVA. Therefore, this easy and fast to perform examination method emerges as a promising visual function endpoint in patients with nAMD and DME in clinical practice and future clinical trials.
